# The Ruggero Ceppellini Advanced School of Immunology and the Neapolitan Scientific Renaissance

**DOI:** 10.3389/fimmu.2019.01494

**Published:** 2019-07-03

**Authors:** Antonio Di Giacomo

**Affiliations:** UOS Haematology, Clinical Pathology Lab, “V. Monaldi” Hospital, Naples, Italy

**Keywords:** school, Serafino Zappacosta, immunology, renaissance, Naples (Italy)

## Abstract

In this article the author, cofounder with Serafino Zappacosta and few other knowledgeable scientists of the Ruggero Ceppellini Advanced School of Immunology in 1991, discusses the significance of this initiative not only for the spreading of immunological culture among scientists—including those from disadvantaged Countries—but also for the resurgence of the city of Naples as a cultural pole of attraction for brilliant minds, as it was in its past history. This is a tribute to Serafino Zappacosta's foresightedness and generosity.

Great intellectual achievements are the excellent fruits of rich and stimulating environments, fertile soils that prepare and nurture the mind. Roses do not bloom in a desert.

In this respect, culture constitutes the background for the development of new ideas and discoveries, that in every field of human knowledge represent the tools of advancement and innovation.

Biological sciences, intended as the study of the significance of the processes of life and not only their mechanical aspects, share with the human sciences the vast realm of the thinking mind, since when man experienced what Teilhard de Chardin defined as “the first moment” of self-awareness. Questions started to appear and science was born.

These and other related considerations was I debating in my thoughts when I met professor Serafino Zappacosta in a gray afternoon of November 1988 in the venues of a course on “Immunity in human pathology” that he used to give every 5 years at the Medical Faculty of the University of Naples “Federico II” where he was tenure professor of Immunology.

Indeed I had known him and his fame from before, as a medical student of that Faculty, always attracted by his charismatic personality and by the matter of his teaching, Immunology, at that time still a fast growing science, but I never had the opportunity of joining his group, nor did I try, the development of my career bringing me elsewhere. On that occasion, however, he showed interest in my curriculum and my recent experience abroad in experimental Immunology, and invited me to give seminars in his Institute about experimental cancer immunology, the subject of my studies. A collaboration started at that point, as did a long friendship.

In those years professor Zappacosta was maturing the intention to create a School of Immunology of international relevance in Naples, that would attract renowned scholars of that matter as teachers and an international audience of students coming from all over the world, in particular from developing Countries, in order to spread and promote immunological knowledge among scientists. He proposed me to join this project, along with a restricted group of scientists such as:

Melchiorre Brai, professor of Immunology at the University of Palermo; Giovan Battista Ferrara, professor of Human Genetics at the University of Naples “Federico II”; Albert Nisonoff, professor of Biology at Brandeiss University, Mass. USA; and Dr. Ciro Manzo, head of the Immunology Department of the Istituto Pascale in Naples. I enthusiastically accepted, of course, honored and flattered by his invitation.

The School was founded in June 1991 with the name “Ruggero Ceppellini Advanced School of Immunology,” dedicated to the memory of Ruggero Ceppellini (1917-88), the great Italian immunogeneticist, as a non-profit scientific association whose aim was to foster, encourage and propagate all aspects of knowledge relating to immunology and associated disciplines (genetics, microbiology, oncology) in the scientific community in Italy and in other Countries, through the promotion of scientific research, continuing education and in-service training.

The School's structure consisted in a Council of Directors and a Scientific Advisory Board, composed by a group of scientists each prominent in different areas of Immunology, that proposed and in turn took charge of the courses each year. Technically, the School teaching programmes were conceived and realized according to a “three level” scheme.

Level I courses, the typical refreshing courses, dealing with the so-called continuing education of medical graduates and designed to update the local practitioners on the recent advances having a bearing on their medical thinking and daily operation.

Level II courses, dedicated to young researchers working in Immunology or related fields, wishing to acquire knowledge of a specific topic within the vast area of Immunology. These are 1-week full-immersion activities, often integrated by workshops and small group discussions. Typical audience of these courses has been represented by Ph.D. students coming from all over the world.

Level III courses, short practical laboratory courses, dealing with recent techniques to be applied in research or even in the clinical laboratory, for small groups of graduates.

The School's inaugural ceremonies were held on 11 October, 1992, at Palazzo Serra di Cassano in Naples, the seat of the Istituto Italiano per gli Studi Filosofici, in the occasion of the School's first course, on the immunology of bone marrow transplantation. Many more courses followed, all successful and attended by students from all over the world, but more remarkable was the returning of Naples as the pole of attraction for scientific knowledge and culture after a long period of oblivion.

And this was the focal point of the all thing, the adventure clear to my mind since the beginning, the basic ideal drive that led me to join professor Zappacosta's dream to bring back to Naples the attention of the world's scientific community. Love for science together with love for our land. Naples certainly deserved this tribute as a recognition of its glorious past and its tradition of pole of attraction for excellent minds and inclusive culture.

This was not unexpected in Neapolitan history but determined by peculiar events, both human and geographical that, I believe, led to make this region of the world a favorable one to become a hub for philosophical and intellectual speculation. And science is, as we all know, fundamentally the result of intellectual and philosophical speculation, nothing else.

The same drive that attracted the divine Vergil to the Neapolitan epicurean school of Chiron, his master and philosophical mentor for masterpieces as the Georgics, led a young and enlightened German emperor in the early Middle Ages to promote Naples as the center of culture, founding the oldest University of Europe and therefore of the western world. The “Studium” established in Naples by Friedrich II Hoenstaufen, emperor and innovator, “*stupor mundi*” as he was called by most historians, was not technically speaking the first one of its kind. The University of Bologna preceded it of many years, but Naples University was the first public institution of a State, born for the political will of a Ruler whose project was that of creating a place where studies were possible without having people leave home, and constituting a center of attraction for scholarly minds. It was an operation of qualified touristic promotion, we might say today, where thegoal was the cultural growth of the place that would become in turn economical and social. At that time is ascribed the myth of the four founders, a Jew, an Arab, a Greek and a Latin, not real individuals but cultural influences concurring to the building of the *ars medica*, and the body of laws that regulate the teaching and the practice of medicine as stated in the “*Liber Constitutionum”* in A.D. 1231. Philosophical speculation and observation of the reality, theory and practice, *ratio et observatio* were the leading criteria for the development of scientific rationales and approaches.

Naples has therefore been since the far past the place where different culture met and merged, creating one of the first melting pots of peoples and ideas in history, favored by its geographical position at the center of the Mediterranean and by the efforts of enlightened rulers, like Friedrich II and, more recently, like The Bourbon kings. Starting with Charles III Bourbon, in fact, Naples became along with Vienna and Paris one of the best and most advanced courts in Europe, both for magnificence of arts and for scientific institutions. The first railroad in Italy between Naples and Portici was built in the year 1836, the first Italian scientific museum of mineralogy was established in the city in 1801 and the first volcanic observatory of the world was built on the slopes of Vesuvius in 1841. Naples was also the place where, in 1872 the eminent German biologist Anton Dhorn built the second laboratory of marine biology in Europe, which hosted, among many others, Ilia Metchnikoff, the second Nobel prize winner for medicine. In Naples took also place the seventh Meeting of the Italian Scientists in 1845, with the participation of 16 hundreds scientists, more than 8 hundred of whom from Naples and the south of Italy. Cultural and scientific growth called for economic growth.

Other times have not been so favorable however. Periods of glory alternating with periods of decline, mainly determined by political instability and short-sightedness have determined the cultural oblivion that has especially characterized the scientific life of the city. It looked as though Naples, in spite of sporadic and meritorious efforts operated at different levels by singular initiatives, substantially relied on its traditional and popular image of a place to visit for touristic reasons. An initiative of restoring the glorious role of the past was at this point needed by many people operating in the scientific field. This led to a dreamer such as Professor Zappacosta to enterprise this initiative of creating a novel cultural start that would bring here the best of the world scientists who would in turn attract again an international qualified audience. The project was also highly philanthropic, since a great deal of attention was dedicated to the students coming from developing Countries who were actively encouraged to attend the courses by granting them bursaries also obtained by international benefactors like the Bill and Melinda Gates Foundation. This was a theme particularly dear to the founders, who envisioned the city to become once again a bridge between the western world and less fortunate areas of the planet that would benefit of this shared knowledge and culture. I recognize in those ideals the reason for my enthusiastic adhesion to Serafino's generous effort and I will be always grateful to him.

The legacy of the Ceppellini School, still vivid in our minds, can be summarized in the motto suggested by Professor Zappacosta “*non multa sed multum*” (“not many but much”, i.e., “quality, not quantity”), that best represents the spirit of an institution that is progressively changing the perception of ourselves and of the role of the western culture in the world.

This perfectly fulfills the prophetic perspective of Serafino Zappacosta for a new era of Immunology and Immuno -Oncology in which a more humanistic and philosophical approach should prevail in research. May the Ceppellini School continue to be a beacon for decades to come.

## Author Contributions

AD is a co-founder of the School of Immunology.

### Conflict of Interest Statement

The author declares that the research was conducted in the absence of any commercial or financial relationships that could be construed as a potential conflict of interest.

